# Does artificial feeding impact neonate growth rates in a large free-ranging mammal?

**DOI:** 10.1098/rsos.221386

**Published:** 2023-03-22

**Authors:** Laura L. Griffin, Amy Haigh, Bawan Amin, Jane Faull, Fiachra Corcoran, Connie Baker-Horne, Simone Ciuti

**Affiliations:** ^1^ Laboratory of Wildlife Ecology and Behaviour, School of Biology and Environmental Sciences, University College Dublin, Dublin 4, Ireland; ^2^ School of Biological Sciences, Queen's University Belfast, Belfast, Northern Ireland

**Keywords:** human–wildlife interactions, feeding interactions, artificial selection, weight, fitness, offspring survival

## Abstract

Variation and disparity in resource access between individuals in an animal population within human-dominated landscapes require attention as artificial selection processes may be at work. Independent, recreational human–wildlife feeding interactions constitute an increasingly prevalent, yet understudied, food resource for birds and mammals living in our cities. However, only a limited number of risk-taking individuals may access it. Using urban fallow deer as our model species, we hypothesized that if these interactions result in positive effects for the engaging individual, e.g. increased milk quality and yield, then this would result in the increased growth rates of their offspring. Alternatively, if these individuals were prioritizing investing time in engagement with humans, resulting in decreased maternal care, then this would result in slower growth rates in their offspring. We found that the offspring of those females that regularly interacted with humans displayed significantly faster growth rates than their risk-adverse counterparts. This advantage for fearless mothers in terms of boosted neonatal growth rates could be mirrored in birds accessing garden feeders, seagulls or pigeons utilizing urban resources, or seals approaching city harbours. Here, we add a new piece to the complex puzzle of how humans are impacting wildlife living within human-dominated landscapes.

## 1. Introduction

Resource utilization in wild populations is of continuous interest to ecologists. In particular, natural variations in behaviour among individuals within a single population and how this can manifest in different ways of using resources have gained significant research attention [1,2]. It has become clear that different behaviours can be associated with different advantages or disadvantages for the individual. For example, in terms of foraging behaviours, animals may either search for food themselves (producing) or follow the food discoveries of other individuals (scrounging). These behaviours have been shown to be repeatable among individuals [2,3]. Notably, producing individuals have immediate access to new, undecimated resources before others arrive (i.e. finder's share) [4,5], while scrounging individuals may be limited on resource intake. However, scrounging individuals perform less dangerous exploratory behaviours, which can provide survival advantages [6], though this may be negated when food is limited [3].

Similar patterns can also be seen in the utilization of urban landscapes: certain individuals show flexible behaviours which enable them to effectively access human-dominated systems in order to scavenge anthropogenic food, while others do not [7,8]. Individuals that use these spaces gain the advantage of additional food sources, but those individuals that choose to avoid these spaces do not experience the resultant conflict with humans [9]. Ultimately, individuals can vary enormously in their approaches to resource acquisition, with some flexible individuals accessing and monopolizing new resources, albeit often in a risky way.

One potential food resource that remains understudied, especially regarding the effects it has on those that utilize it, is that of independent, recreational human–wildlife feeding interactions. This is the process by which humans hand-feed both marine (e.g. seals, dolphins) and terrestrial (e.g. seagulls, ducks, foxes and deer) wild animals outside of controlled environments and without being monitored. Notably, similar to the results seen in the aforementioned urban studies, it has been shown that not all individuals in a population will engage with these interactions. In fact, likelihood to interact falls on a spectrum of repeatable among-individual differences, ranging from those that consistently approach people for food to those that rarely, if ever, do so [10]. This variation in engagement among individuals means that there are very different dietary intakes across a single population, i.e. those that consume more natural diets and those who are supplemented by these interactions. As this food is offered independently of management or conservation-informed provisioning by laypeople, items may include many high-sugar, human snacks [10]. This raises concerns about how this food could be affecting the individuals involved. It also marks these interactions as a potential driver of artificial selection if (i) they provide either fitness advantages, or prove to be detrimental, to the subset of individuals that access them and (ii) if this behaviour is indeed heritable [11,12].

Previous studies have shown that supplementing the diet of animals with additional food can improve milk production in lactating females [13], increase *in utero* productivity in pregnant females [14,15], and improve overall body condition [16]. This, ultimately, provides evidence that this additional feeding improves female fitness and productivity. However, these results come from studies under controlled conditions and/or where appropriate foods are carefully provisioned. By contrast, other studies have reported that feeding can lead to protein deficiencies [15] and decreased maternal care, resulting in infant mortalities [17], particularly in situations where the food provided is not monitored or controlled. This calls into question what the true effects of independent human–wildlife feeding interactions are on the wildlife involved. Notably, it is extremely difficult to track changes in lactation and pregnancy in free-living mammals; however, the resultant impacts on offspring are more accessible as a source of data.

Here we aim to explore whether these independent feeding interactions are either beneficial or detrimental to the wild individuals that engage with them in terms of the early-life growth rates of their offspring. Variations in growth rates represent the associated impacts on milk yield, quality and intake in mammals [18–21]. Notably, if the acceptance of food from these interactions results in increased growth rates, and therefore the increased weight of associated offspring, then this could provide survival advantages for said offspring [22–25]. In other words, these feeding interactions would be directly promoting the fitness of those females involved. We, therefore, hypothesize that, if feeding has an effect on the individual, it will manifest as differences in early-life growth rates between the offspring of those females that accept food handouts from humans and those that do not. If females do gain an advantage by engaging in these interactions, due to increased nutritional intake, then this would result in increased growth rates in their offspring (H_1_).

While we have previously demonstrated that the offspring of mothers that regularly receive food from humans have higher weights at birth [10], studies have shown that engagement with these interactions can result in decreased maternal care, as mothers invest large amounts of time in pursuing humans for food [17]. For this reason, it could be hypothesized that the offspring of interacting mothers would show decreased growth rates during the early stages of life, if mothers in this population mimicked similar reductions in maternal care. This would mean that the offspring of non-interacting mothers would have comparatively higher growth rates and would, therefore, close the gap that existed at birth (H_2_).

## 2. Methods

### 2.1. Study area and population

The study was performed in Phoenix Park, Dublin, Ireland; a 7 km^2^ urban park that is a popular destination for both international tourists and local visitors. In the last decade, feeding the resident fallow deer (*Dama dama*) population of approximately 600 free-ranging individuals has become a popular activity with visitors. Circa 80% of these deer are individually tagged with unique ID ear-tags, which have been placed annually during the routine fawn-tagging performed by University College Dublin wildlife biologists since the 1970s. These tags allow for observational data to be easily collected as part of the ongoing monitoring and management of the population [10,26]. Notably, deer numbers are maintained at the park's carrying capacity through annual winter-time culls, which are led by the Office of Public Works.

### 2.2. Data collection—begging ranks

Studies in this site had previously shown that the vast majority of the individually recognizable (i.e. ear-tagged) deer in this population can be classified along a continuum of likelihood to interact with humans. This ranges from individuals that consistently approach people for food, a behaviour referred to as ‘begging’, to those that rarely, if ever, do so. This individual classification is referred to as their ‘begging rank’ [10].

We collected observational data on individual presence and engagement in feeding interactions for our female population over 4 years (2018–2021) during the summer months (May–July in 2018 and 2019, and June–July in 2020 and 2021). This time period was selected as it encompasses the final weeks of gestation, the birthing period (i.e. mid-June), and the first weeks of life for neonates. The begging behaviour of the females that were observed each year was then modelled as outlined in Griffin *et al*. [10], with each year being modelled individually. A description of the method used and adapted for this work has been included in the supplementary material (electronic supplementary material, S1). Their begging ranks, which are composed of the best linear unbiased predictors (BLUPs) [27] produced by the models, were then extracted using the *ranef* function in the *lme4* package [28,29].

### 2.3. Data collection—fawn weights

As fallow deer adopt a ‘hider’ form of anti-predator strategy, fawns are isolated from conspecifics and hidden in vegetation during their first weeks of life. During this time, they are regularly visited by their mother to be nursed before they start to join the herd at approximately two weeks old [30,31]. This hider mechanism means that these isolated offspring can be easily captured during this brief period, enabling the direct collection of physiological data and the ear-tagging of these fawns. We performed these collections in June over the same 4-year period (2018–2021) as the female data were collected. Captures were performed as outlined by Amin *et al*. [30].

Once captured, we recorded the fawn's sex and then estimated its age (in days) by assessing the condition and wear of shoes and dew claws and by examining the length and condition of the umbilical cord (*sensu* [30]). We then placed the fawn in a 100 l bag and weighed it prior to release, with the weight being documented in kilos. If the fawn was spotted again after approximately 48 h or more had passed, then we recaptured it and documented the age and weight again. A fawn was permitted to be captured a maximum of four times in total (i.e. the initial capture and three recaptures) after which point the team avoided disturbing the fawn again in order to avoid over-handling.

### 2.4. Data collection—mother–fawn pairing

Over the same 4 years, we collected observational data pairing ID'd mothers with their offspring from July–September (i.e. once the fawns had begun to join the herd out in the open). We walked the side of the park that is mainly used by female deer, along with some subadult males, (i.e. the western side of the park, occurring due to natural sexual segregation [32]) until a herd was located, at which point we began observations. We recorded three different types of interactions between does and fawns: (i) suckling by the fawn, in which we clearly distinguished between allosuckling (from the back; not counted) and frontal suckling (where the doe can see the fawn), (ii) social grooming between the doe and the fawn, and (iii) following behaviour, where the fawn sticks close to the doe. To limit incorrect pairing, we only confirmed a mother–fawn pair if the same pair had at least two independently observed interactions.

### 2.5. Dataset formation and analysis

We merged these datasets to form a final set consisting of 278 rows, with each row representing either a first capture or a recapture of a fawn [33]. Each row included the ID of the captured fawn, the age of the fawn at the capture (in days), the sex of the fawn, the year of its birth (i.e. the year it was tagged), the ID of its mother, the exact age of the mother (calculated by deducting the year of her birth, which we had from previous fawn-tagging records, from the year of her fawn's birth), and the mother's begging rank. This final dataset included 170 fawns across 107 mothers, of which 27 mothers were paired with a fawn in only one year and 80 mothers appeared over multiple years. Of the 170 fawns, 91 were captured once, 53 captured twice, 20 captured three times and six captured four times. In terms of the number captured per year: 36 fawns were captured in 2018, 36 fawns in 2019, 46 fawns in 2020 and 52 fawns in 2021.

**Figure 1.**
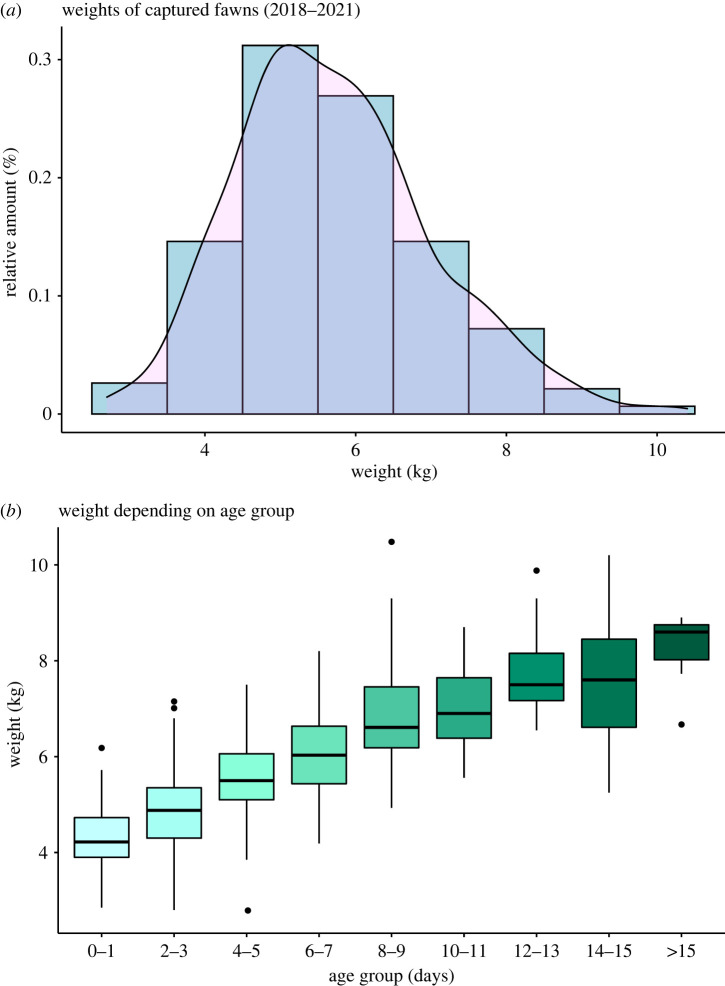
Plots depicting the distribution of weight at capture for neonatal fallow deer (*Dama dama*) fawns over 4 years (2018–2021) (*a*) and weight of those same fawns depending on age in days (*b*) in Phoenix Park, Dublin.

We fit two linear mixed effects models (LMMs) following *a priori* structure, with the fawns' weight at capture as the response variable, using the *lmer* function in the *lme4* package [28] for both models. Both models also included the ID of the fawn, the ID of the mother and the year of capture as crossed random effects. In terms of predictors, we included the age of the fawn at capture/recapture (days), the age of the mother (years), the begging rank of the mother and the sex of the fawn (categorical: male, female). The age of the fawn was included as we expected the fawn's weight to change as it aged (figure 1 for graphic representation of fawns' weight at capture in this particular site).

The mother's age was included, as older mothers would have had greater numbers of offspring over previous breeding seasons, and this experience could inform mothering behaviours, providing their offspring with advantages [34]. The begging rank of the mother was included as we had previously identified that mothers with higher begging ranks produce heavier fawns at birth [10], and the sex of the fawn was included as male fawns tend to be larger than female fawns [34,35]. Note that the fawns captured once in the database contributed to our understanding of the relation between mothers' begging rank and fawns’ weight at birth, i.e. these data improved our estimate of the intercept of the model (weight of the fawns when their age is 0 days old), whereas fawns captured multiple times contributed to our understanding of growth rates as a function of the age of the fawn. We included a two-way interaction in each of the models between the age of the fawn and the begging rank of the mother, as we wanted to test whether fawns gained weight differently over time across different begging ranks (i.e. our main hypothesis).

All numerical predictors were scaled to improve model convergence. The only difference between our two *a priori* models was that one model (M1), included all fixed effects as both single and quadratic terms to allow for nonlinear patterns. However, only the linear predictors were included in the second model (M2). Goodness of fit metrics were then extracted for both models, using the *r.squaredGLMM* function in the *MuMIn* package [36]. Both *a priori* models were then compared using Akaike information criterion (AIC), and the one with the best (i.e. lowest) AIC was selected as the optimal model. Models were fitted using maximum-likelihood (ML) estimation when extracting AIC for model comparison, whereas they were refitted using restricted maximum-likelihood (REML) estimation to report and interpret parameter estimates [37]. Prior to modelling, predictors were successfully screened for collinearity (|*r*_p_| < 0.7, [38]).

Once our preferred model, i.e. the one with lower AIC, was identified, significant model predictions were plotted with marginal 95% confidence intervals using the *effects* library [39]. Due to debate regarding the use of BLUPs [40,41], we reran the lower AIC model using a categorical variable computed using the errors generated by the model without any changes in results (*sensu* [10]) (see electronic supplementary material, S2). We also reran this model after excluding those fawns that were captured only once (see electronic supplementary material, S2). In both cases, model results and patterns were similar to those achieved with the main model, confirming the validity of the main model presented here.

## 3. Results

M2 (i.e. the model using only single fixed effects, AIC: 540.648) was selected as the preferred model due to its lower AIC compared with M1 (i.e. the model using both single and quadratic effects, AIC: 542.961). M2 explained 93.34% of the variation of the model, with 64.46% being explained by the fixed effects alone. All single predictors were flagged as significant, as well as the interaction (table 1). The effects for M1 are available in the supplementary material (electronic supplementary material, S3) and show similar effects in all aspects except that mother age was not flagged as significant. This is probably due to masking effects resulting from this more complex model.

**Table 1.** Parameters estimated by the linear mixed-effects model, including single fixed effects alone (M2), explaining the variation in weight in neonate fallow deer (fawns) in Phoenix Park, Dublin (2018–2021) as a function of fawn age (growth rate) and other confounding factors. The model was fitted on 278 captures of 170 individually ID'd fawns. These 170 fawns were produced by 107 different ID'd mothers, with some mothers having fawns across multiple years. Year, ID of the mother and ID of the fawn (Tag) were fitted as crossed random intercepts. Italics indicate significant *p*-values.

fixed effects	estimate	std. error	*t* value	Pr(>|*t*|)
intercept	5.593	0.104	53.806	*<0.001*
age of the fawn (days old)	0.940	0.026	36.828	*<0.001*
age of the mother (years old)	0.220	0.066	3.352	*0.001*
begging rank of the mother	0.136	0.061	2.238	*0.027*
sex of the fawn [female]	0	—	—	*—*
sex of the fawn [male]	0.396	0.114	3.486	*<0.001*
age of the fawn × begging rank of the mother	0.065	0.030	2.217	*0.028*

**Figure 2.**
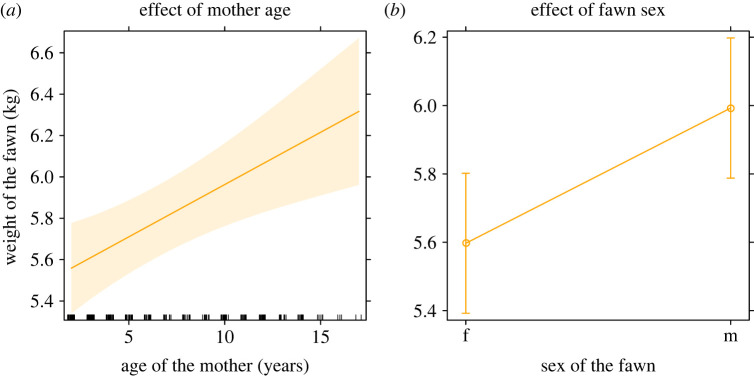
Plots depicting the effect of mother age (*a*) and fawn sex (*b*) on the weight of neonatal fawns in Phoenix Park, Dublin, as predicted by a linear mixed-effect model (LMM) M2 (i.e. the model using single, linear predictors only). Predicted effects are shown with the marginal 95% confidence intervals.

**Figure 3.**
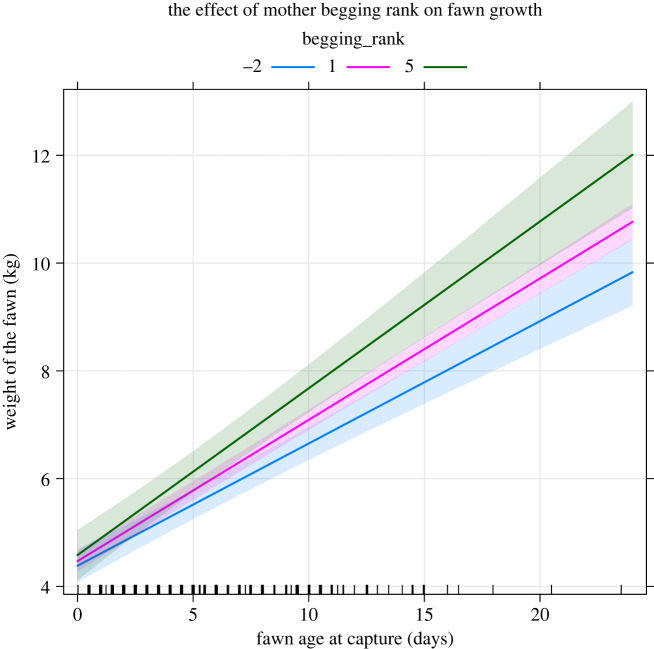
Multi-line plot depicting the differences in growth rate over time for the offspring of mothers with different begging ranks (i.e. BLUPs predicting individual likelihood to interact with humans for food) in Phoenix Park, Dublin, as predicted by a linear mixed-effect model (LMM) M2 (i.e. the model using single, linear predictors only). Predicted effects are shown as lines with the marginal 95% confidence intervals.

For M2, fawn birthweight increased with the age of the mother (figure 2*a*) and male fawns were significantly heavier than female fawns (figure 2*b*). Additionally, the interaction was flagged as having a clear effect, with 95% confidence intervals not overlapping zero (table 1). This means that the offspring of mothers with higher begging ranks both stayed consistently heavier than those of mothers with lower begging ranks and also gained more weight over time (i.e. grew faster) (figure 3).

## 4. Discussion

The goal of this study was to identify whether engaging in recreational human–wildlife feeding interactions results in advantages or disadvantages for females of a free-ranging wild mammal population, as indicated through the early-life growth rates of their offspring. Here, for the first time, we have identified that the offspring of female deer that consistently accept food from people have significantly faster growth rates during their first weeks of life than those fawns whose mothers do not interact (H_1_). While the risk of decreased maternal care in favour of investing time in these interactions [17] suggested that these offspring may grow significantly slower, our study has shown that the opposite mechanism is in effect, ergo rapidly widening the gap in weight between the offspring of those that regularly engage in interactions and other individuals' offspring. This indicates that these mothers are utilizing this human food source to extract additional nutrition compared with those mothers that remain on a natural diet, potentially improving their milk quality and yield [13]. With improved milk yield and nutrition intake from human foods, there is also the potential that these mothers can invest more time in nursing offspring, resulting in these increased growth rates. Notably, all mothers had previously been identified as being present in the same herds and with access to the same resources, leaving engagement with these interactions as the only dietary variable [10].

In regards to the confounding factors included in our sampling design, the age of the mother and the sex of the fawn have also been identified as factors affecting the weight of fawns in this study. Fawn weight increased with mother age, a factor that has previously been identified in other sites [42,43]. This is probably due to maternal experience as well as the fact that maternal mass strongly influences birth mass, so this may be expected to be higher in prime females [43,44]. The fact that male fawns were typically heavier than female fawns is also supported by literature regarding inter-sexual variations in birthweights in cervids [35]. These findings are, therefore, consistent with previously established research, supporting the robustness of our study. This marks our novel findings regarding the effects of mother engagement with feeding interactions on the weight and growth of offspring as of significant interest.

Heavier weights are associated with survival advantages in mammals [22–25], including in our own study site [45]. For example, young individuals are more likely to survive winter periods of decreased forage if they are heavier [46] and are less likely to be predated early in life [25,43]. Additionally, heavier weights and faster growth rates in the early stages of life are associated with larger body sizes in adulthood [47,48]. Larger body size in adulthood is linked with greater mating success for male cervids [49] and greater fecundity for female cervids [50]. This indicates that those females who are interacting with humans for food may have increased maternal fitness as a result (yet to be tested). Likelihood to interact with people for food is a repeatable behavioural trait [10] probably associated with boldness, similarly to those individuals that opt to utilize urban, human-dominated landscapes as a food source [51]. If this behaviour is heritable [11,12] then artificial selection may be at work and it could become more predominant in populations experiencing these direct human interactions. It is also possible that mothers interacting with people, i.e. bolder individuals, generally are improved foragers that invest greater time in food acquisition with reduced vigilance. This would mean that these females may have an inherent advantage in terms of resource intake, but that is being further driven to higher, artificial levels due to the consumption of this human food.

This is the first time that variation in early-life growth rates depending on mother engagement with independent, recreational human–wildlife activities has been identified. Utilization of this food source, at the expense of risky, direct contact from humans, is clearly benefiting females within this population. It is likely that this is also occurring within other populations of species or in other areas, resulting in artificial selection of these adaptable, bold behavioural traits. However, there is the potential that effects may vary enormously depending on the types of food being offered, the regularity of the offerings, and the species involved. We, therefore, recommend similar studies be performed across a wide variety of species and study sites to further unravel these effects. Additionally, populations that are regularly exposed to these interactions need to be closely monitored to determine what the long-term impacts are. For example, if artificial selection is at work, and the behavioural type associated with interactions becomes more prevalent, then there could be associated impacts on population viability and plasticity [52,53].

We encourage wildlife managers to consider the potential implications that these findings hold for associated management activities. These interactions occur across many species in a variety of areas, including urban areas [54], urban parklands [10,55] and national parks [56]. Notably, management of these areas is typically the responsibility of governmental groups, local councils, and park officials. Our findings may, therefore, inform management of recreational, self-motivated feeding interactions in these areas and by these groups. For example, wildlife in these areas are often managed through systematic culls in order to maintain populations at regulated numbers [57,58]. If the provision of food to these wild individuals by humans is resulting in advantages for select individuals and the increased survival of their offspring [10,45], then there may potentially be associated population explosions that would require more regular and costly culling actions. If this is the case, then effective management of these feeding activities may prevent the need for increased culling before it even occurs. This would be invaluable for public relations between the local communities and park managers, as culling activities are already recognized as a controversial topic for members of the public [59,60], but this is something that requires further exploration.

Similarly, management geared towards maintaining the health and safety of park visitors is of high priority to site managers, particularly where engagement with wildlife in concerned [61]. This is because the proximity that is required to engage in activities with wildlife, such as direct recreational feeding interactions, already puts people at risk of injury [62,63]. If this begging behaviour is transmitted from mother to offspring and these offspring have a better chance of survival, then our research indicates that this behaviour is being artificially selected for by these feeding activities. This would mean that this bolder, begging behavioural type could become more predominant in wild populations over time and that the risk of injury to humans could also increase; something that will also need to be explored in future studies and management actions.

Finally, it is important to note that our research indicates that a subset of this wild population is gaining a significant advantage over other individuals that are ingesting a more ‘natural’ diet. This may be perceived as feeding interactions being beneficial for wildlife, but this may vary depending on the types of food that are being offered, as mentioned above. For example, there may be periods where more digestible foods are offered, and times where indigestible foods become popular, potentially due to public misconceptions about the diet of the targeted wildlife species. We, therefore, emphasize the need for wildlife managers to consider how potential impacts may vary between sites depending on the types of foods offered by visitors, and how the non-interacting subset of these populations may be disadvantaged, as opposed to simply assuming that these activities always result in positive gain for the wildlife involved.

## Data Availability

Data are available on Dryad Digital Repository: https://doi.org/10.5061/dryad.sbcc2fr9r [33]. The data are provided in electronic supplementary material [64].
